# Characterization of Influenza Hemagglutinin Interactions with Receptor by NMR

**DOI:** 10.1371/journal.pone.0033958

**Published:** 2012-07-16

**Authors:** Christopher McCullough, Minxiu Wang, Lijun Rong, Michael Caffrey

**Affiliations:** 1 Department of Biochemistry and Molecular Genetics, University of Illinois at Chicago, Chicago, Illinois, United States of America; 2 Department of Microbiology and Immunology, University of Illinois at Chicago, Chicago, Illinois, United States of America; Università di Napoli Federico II, Italy

## Abstract

In influenza, the envelope protein hemagglutinin (HA) plays a critical role in viral entry by first binding to sialic acid receptors on the cell surface and subsequently mediating fusion of the viral and target membranes. In this work, the receptor binding properties of influenza A HA from different subtypes (H1 A/California/04/09, H5 A/Vietnam/1205/04, H5 A/bar-headed goose/Qinghai/1A/05, and H9 A/Hong Kong/1073/99) have been characterized by NMR spectroscopy. Using saturation transfer difference (STD) NMR, we find that all HAs bind to the receptor analogs 2,3-sialyllactose and 2,6-sialyllactose, with subtle differences in the binding mode. Using competition STD NMR, we determine the receptor preferences for the HA subtypes. We find that H5-Qinghai and H9-Hong Kong HA bind to both receptor analogs with similar affinity. On the other hand, H1 exhibits a clear preference for 2,6-sialyllactose while H5-Vietnam exhibits a clear preference for 2,3-sialyllactose. Together, these results are interpreted within the context of differences in both the amino acid sequence and structures of HA from the different subtypes in determining receptor preference.

## Introduction

The membrane glycoproteins hemagglutinin (HA) and neuraminidase (NA) play critical roles in influenza infection [1]. For influenza A, the cause of seasonal flu, the antigenic properties of HA and NA are used for classification into subtypes (HA: H1–16 and NA: N1–9) with H1N1 and H5N1 being of particular concern. For example, the H1N1 influenza pandemic of 1918 resulted in over 50 million deaths worldwide and, despite improved vaccination efforts and better treatments, seasonal influenza is still responsible for greater than 250,000 deaths per year worldwide [2], [3]. Moreover, the highly pathogenic avian influenza H5N1, which is rarely transmitted to humans, has a >60% fatality rate and is regarded as a potential pandemic strain [4]. Current treatments for influenza include Tamiflu (oseltamivir) and Relenza (zanamivir), which target NA [5]; however, drug resistance is an ongoing concern. Indeed, the 2008–2009 H1N1 strain exhibited ∼100% resistance against Tamiflu [6], and thus influenza may now be considered as a drug-resistant pathogen.

HA, as well as analogous envelope proteins from Ebola, HIV, and sialic acidRS-CoV, mediates virus entry through binding to receptor and conformational changes that result in fusion of the viral and target cell membranes [7], [8], [9]. HA is synthesized as a precursor, HA0, which is subsequently cleaved to form a complex consisting of HA1, the receptor binding subunit, and HA2, the subunit that mediates membrane fusion [1], [10]. In the first step of infection, HA trimers bind to sialic acid receptors on the target cell surface. In the second step, the virus enters the endosome and the resulting low pH triggers a large conformational change in HA, exposing a hydrophobic region, termed the fusion peptide. The newly exposed fusion peptide then inserts into the target membrane, thereby bringing the viral and target membranes in close contact and allowing membrane fusion and entry of the virus core into the cytoplasm [7].

Due to its critical nature, the initial step of influenza entry, HA binding to sialic acid, has received much attention [7]. HA binds to sialic acid possessing either α2,3- or α2,6-linkage to galactose. Interestingly, receptor preferences are thought to define host range [11]. For example, in avian species α2,3-linked receptors are most abundant in the digestive tract, the primary site of influenza infection in avians, and in humans α2,6-linked receptors are most abundant in the upper respiratory tract, the primary site of influenza infection in humans [12], [13]. Therefore it is of interest to consider the receptor usage, particularly for the highly pathogenic avian strains such as H5. Early detection of subtype H5 HA that bind to α2,6-linked receptors may be used to efficiently deploy vaccination or treatment strategies. Moreover, the receptor binding site of HA, which determines receptor preference, is the target for neutralizing antibodies, as well as an attractive target for therapeutic intervention. Consequently, a better understanding of mutations that occur within the receptor binding site may be useful in vaccine and drug design efforts. In this work, we have used NMR spectroscopy to better define the sialic acid interactions with HA from subtypes H1, H5 and H9. We find that H5-Qinghai and H9-Hong Kong HA bind to the receptor analogs 2,3- and 2,6-sialyllactose with similar affinity. In contrast, H1 exhibits a clear preference for 2,6-sialyllactose while H5-Vietnam exhibits a clear preference for 2,3-sialyllactose. Based on the previously determined high-resolution structures of the HA studied, we will discuss the determinants of receptor preference.

## Results

### HA Subtypes H1, H5 and H9 Bind to 3′SL and 6′SL with Relatively Low Affinity

**Figure 1:**
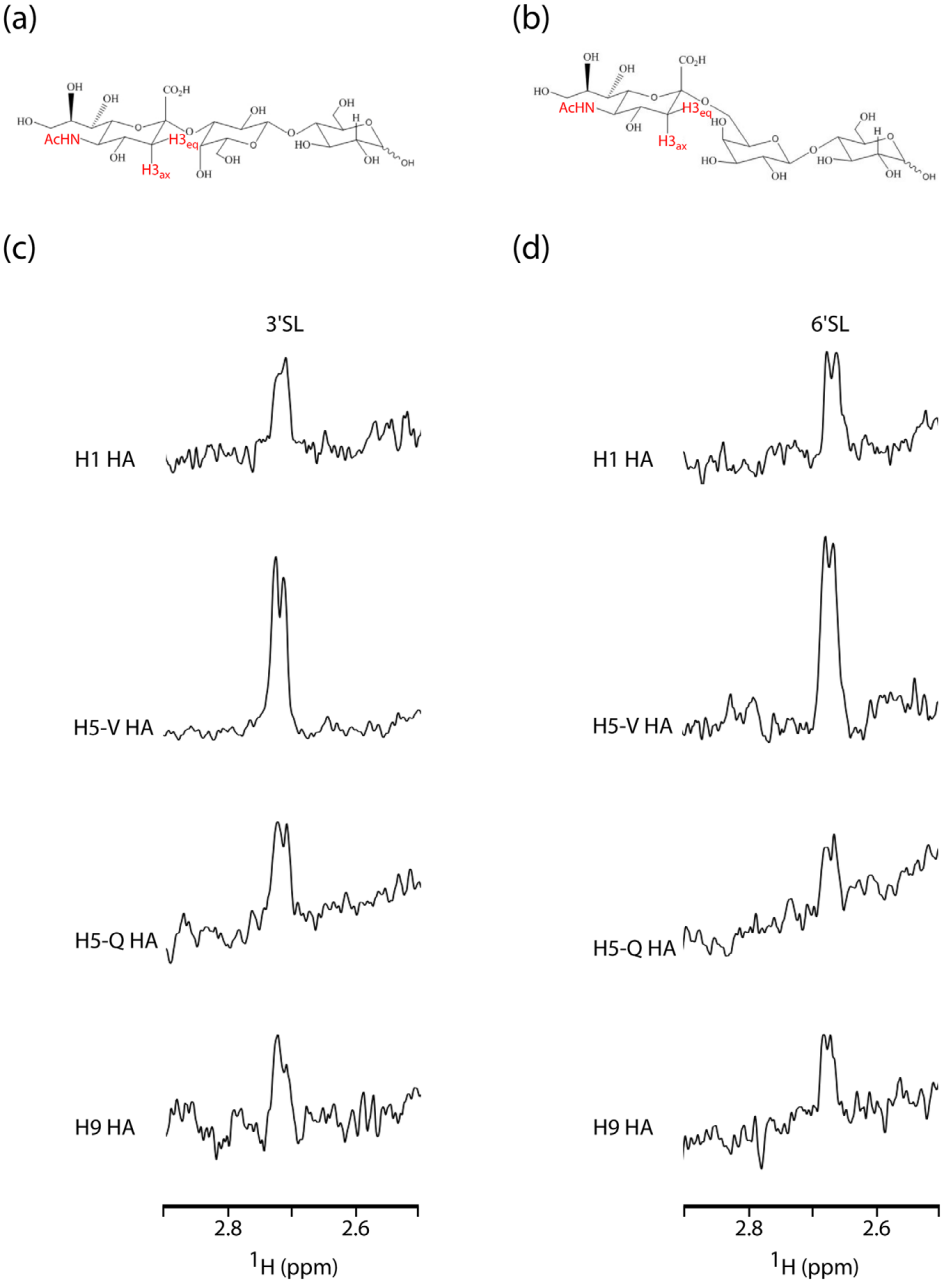
STD studies of 3′SL and 6′SL binding to HA from subtypes H1, H5 and H9. (a) 3′SL chemical structure. (b) 6′SL chemical structure. (c) STD of 3′SL binding to HA from subtypes H1, H5 and H9. (d) STD of 6′SL binding to HA from subtypes H1, H5 and H9. The ^1^H to be used as reporters are denoted by arrows in (a) and (b). For (c) and (d) the experimental conditions were 2 µM HA, 3 mM 3′SL or 3 mM 6′SL in PBS, pH 7.4 at 25°C with an identical number of scans.

**Figure 2:**
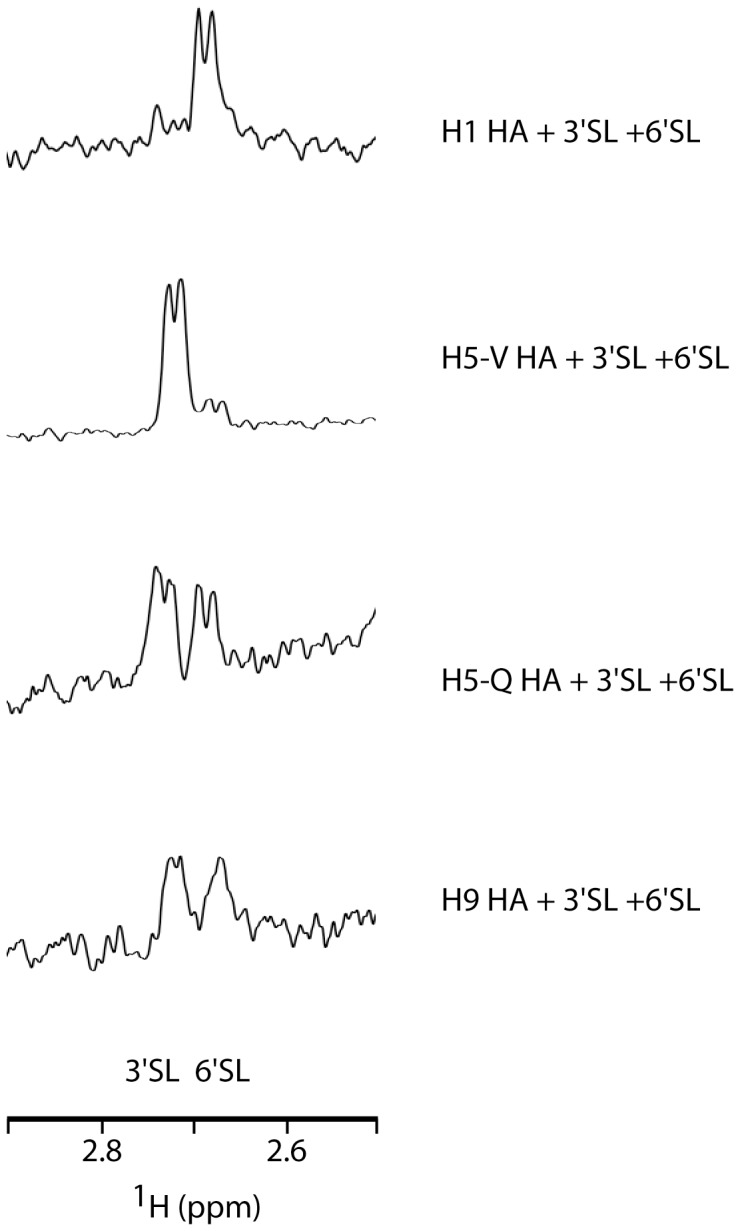
Competition STD experiments for 3′SL and 6′SL binding to HA from subtypes H1, H5 and H9. Experimental conditions were 2 µM HA, 3 mM 3′SL, and 3 mM 6′SL in PBS, pH 7.4 at 25°C with an identical number of scans.

In this work we characterize the receptor binding properties of the HA from three different subtypes of influenza A: H1, H5 and H9. The H1 HA was from a human source of the recent 2009 outbreak, which represents the first pandemic influenza outbreak of the 21st century. The H5 HA were from a human source, H5 HA/Vietnam (hereafter referred to as H5-V), and an avian source, H5 HA/Qinghai (hereafter referred to as H5-Q), both of which represent highly pathogenic H5 isolates. The H9 HA was from a human source of the 1999 outbreak. We chose to use 2,3-siallylactose (3′SL, Figure 1a) and 2,6-siallylactose (6′SL, Figure 1b), which are analogs to the human and avian receptors, respectively [14], to probe HA receptor preference. Note that more complex oligosaccharides give rise to overlapping signals that are difficult to interpret in NMR experiments [14]. To characterize interactions of the receptor analogs with HA, we used the technique of STD NMR. In the STD NMR experiment, the resonances of the large molecule ^1^H are selectively irradiated (HA in this case), and subsequently magnetization is transferred to the ^1^H of small molecules (the SL) that exchange between bound and free states during the irradiation period; the difference, with respect to a reference spectrum in which the large molecule ^1^H are not irradiated and hence no magnetization transfer occurs, identifies ^1^H of the small molecule that are in closest contact to the large molecule in the bound state [15], [16], [17]. In Figure 1c and 1d we present a region of the STD spectrum for which 3′SL and 6′SL are easily distinguishable (the STD spectrum of the control experiments in which no HA is present is shown in Figure S1). For example, the 3′SL H_3eq_^1^H resonates as a doublet at 2.73 ppm and the 6′SL H_3eq_^1^H resonates as a doublet at 2.67 ppm and thus 3′SL and 6′SL binding are readily determined by the H_3eq_ resonance. From Figure 1c and 1d, it is clear that all 4 HA bind to 3′SL and 6′SL, albeit at relatively low affinity (the experiments were performed at a SL concentration of 3 mM to achieve appropriate signal to noise; little or no binding was apparent at 300 µM ligand concentration). Note that the experiments were performed under identical experimental conditions near the expected K_d_ of ∼3 mM [14] and thus the differences in signal/noise are taken to be due to differences in affinity for the receptor analogs, as well as differences in the binding mode (discussed below, cf. [16], [17]).

### Detection of Differences in the Binding Interactions

In the STD NMR experiment, the relative intensity of ^1^H within a particular ligand gives insight into their proximity to the protein interaction surface [16], [18]. In the case of 3′SL and 6′SL, there are three easily distinguishable NMR signals in the STD spectrum: H_3eq_ (discussed above), H_3ax_, and the N-acetyl group (cf. Figures 1a and 1b). The three reporters are found on the sialic acid moiety of SL, which is the primary site of interaction with HA [7]. Due to differences in the apparent affinity for each HA:SL interaction, the %STD, which is presented in the Table S1, cannot be used to compare one set of interactions to another. Consequently, in Table 1 we show the relative ratio of intensities for the three reporters for a particular experiment. The relative STD of the HA reporters fall into three classes. For example in the cases of H1∶3′SL and H1∶6′SL, the relative STD of the 3 reporters are similar, suggesting that they are similar distances from the HA surface in the bound state. In contrast, in the cases of H5-V:3′SL, H5-V:6′SL, H9∶3′SL and H9∶6′SL, the H_3eq_ and H_3ax_^1^H exhibit significantly lower relative STD than the acetyl ^1^H, suggesting that H_3eq_ and H_3ax_ are more distant from the HA surface in the bound state. Finally, in the cases of H5-Q:3′SL and H5-Q:6′SL, the H_3eq_^1^H shows significantly lower relative STD than the acetyl and H_3ax_, implying that it is somewhat more distant from the HA surface in the bound state. Taken together, the STD NMR experiment suggests subtle differences in the binding mode of sialic acid with the various subtypes.

**Table 1:** Relative ratio of STD intensity for HA interactions with 3′SL and 6′SL^1^.

Interaction	H_3eq_	H_3ax_	Acetyl
H1∶3′SL	**0.8**	**0.9**	**1.0**
**H1∶6′SL**	**0.6**	**0.7**	**1.0**
**H5-V:3′SL**	**0.5**	**0.5**	**1.0**
**H5-V:6′SL**	**0.3**	**0.3**	**1.0**
**H5-Q:3′SL**	**0.2**	**0.7**	**1.0**
**H5-Q:6′SL**	0.2	1.0	0.5
H9∶3′SL	0.3	0.3	1.0
H9∶6′SL	0.2	0.3	1.0

1 The H_3eq_, H_3ax_ and Acetyl resonances of 3′SL occur at 2.73, 1.77 and 2.00 ppm, respectively. The H_3eq_, H_3ax_ and Acetyl resonances of 6′SL occur at 2.67, 1.70 and 2.00 ppm, respectively. The relative ratio of STD intensity is defined as %STD/%STD_max_ for a particular HA interaction, where %STD_max_ corresponds to the largest observed %STD.

### Relative Binding Affinity of HA for 3′SL and 6′SL

In the next step, we performed a STD-based competition assay to determine receptor preference [18], [19]. As discussed above, the H_3eq_ resonance is unique for each type of SL (Figure 1c and 1d). Accordingly, the competition assay shown in Figure 2 uses equal concentrations of the SL in the presence of a particular HA to distinguish relative affinity of the HA for the SL. In Figure 2, the presence of both signals with similar intensity for H5-Q and H9 HA suggests that the HA binds to 3′SL and 6′SL with similar affinities. On the other hand, H1 HA clearly binds to 6′SL with higher affinity based on the significantly higher signal of the 6′SL H_3eq_^1^H (Figure 2). In contrast, H5-V HA clearly binds to 3′SL with higher affinity as suggested by the significantly higher signal of the 3′SL H_3eq_^1^H (Figure 2). Based on the approximation that the ratio of the STD in the competition experiment is proportional to the ratio of the K_d_ at SL concentrations near K_d_ (see Materials and Methods S1), we estimate that the K_d_^3′SL^/K_d_^6′SL^ for H1, H5-V, H5-Q and H9 HA are ∼3, 0.2, 1 and 1, respectively. Finally, we note that in the case of H5-Q HA, we performed an additional experiment in the presence of a 2× higher concentration of 3′SL and observed a decrease in the intensity of the 6′SL signal, consistent with the notion that the binding sites of 3′SL and 6′SL are overlapping (Figure S2).

## Discussion

### HA Subtypes Bind to 3′SL and 6′SL Receptor Analogs

Interestingly, the HA from all three subtypes examined, including those from the highly pathogenic H5, bind to both 3′SL and 6′SL as assayed by NMR. As noted above, HA evolves to utilize receptors found in the target organism (i.e. 3′SL in the avian digestive tract and 6′SL in the human upper respiratory tract, [20]. The ability of HA to bind both types of sialic acid may be a factor in the ability of influenza to switch hosts or indicate that such a strain has not yet adapted to humans [21]. Moreover, the binding of HA to α2,3- and α2,6-linked sialic acid may be particularly relevant in humans in that α2,3-linked sialic acid are found in the lower respiratory tract [22]. Alternatively, the decreased binding affinity of HA for α2,3-linked sialic acid may reduce the inhibitory effects of respiratory mucins, which are rich in α2,3-linked sialic acid [23].

### Receptor Preference of HA

The STD competition experiments shown in Figure 2 establish the relative affinity of HA for α2,3- and α2,6-linked sialic acid, with the caveat that sialyllactose was used as a receptor analog. We observed that H1 binds to 6′SL with higher affinity and H5-V binds to 3′SL with higher affinity, which is in agreement with previous studies using glycan arrays [24], [25], [26], [27]. On the other hand, we observed that H5-Q and H9 bind to 3′SL and 6′SL with similar affinities. Interestingly, Nicholls et al. [13] found that H5 HA is able to infect cells not containing 3′ SL, consistent with our observation that H5 binds to both 3′ and 6′SL. Moreover, Saito et al. [29] found that H9 exhibits α2,3- and α2,6-linked binding based on a hemagglutination assay, an observation in agreement with our results for H9.

### HA Structural Determinants of Sialic Acid Specificity

**Figure 3:**
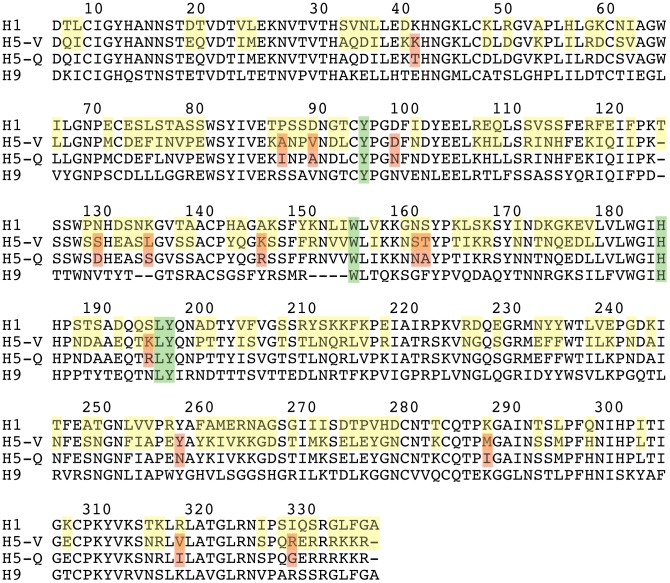
Amino acid sequence alignment for the HA1 of subtypes used in the present work. The numbering corresponds to that of H1 A/California/04/09 HA1. Conserved residues that form interactions with sialic acid [30] are colored green. Non-conserved residues between H1 and H5-V HA are colored yellow. Non-conserved residues between H5-V and H5-Q are shaded red.

Finally, it is of interest to consider the structural determinants for sialic acid specificity. In Figure 3, the amino acid sequence alignment for the four HA that were characterized in the present study is shown. With respect to H1 HA, the sequence identities of H5-V, H5-Q and H9 are 54%, 53% and 43%, respectively. In this figure, five highly conserved residues, Y97, W156, H186, L197, and Y198 (H1 HA numbering), which form direct contacts with sialic acid [30] are shaded green. As presented above, H1 HA exhibits significantly higher affinity to α2,6-linked sialic acid found in humans and H5-V exhibits significantly higher affinity to α2,3-linked sialic acid found in avians. Clearly, the different receptor usage must be due to differences in amino acid sequence (and hence structure). In Figure 3, the differences between the amino acid sequences of the H1 and H5-V strains are highlighted in yellow. Previous work has suggested that residues T139, D193, S196, E219, P224, K225, D228, Q229, E230 and G231 of H1 HA (H1 HA numbering) are implicated in receptor specificity [31]. With respect to the HA characterized in the present study, K225, Q229 and G231 are identical in H1 and H5-V and thus not determinants of receptor specificity in our case. On the other hand T139, D193, S196, E219, P224, D228 and E230 of H1 HA are substituted by S139, E193, K196, R219, S224, G228 and S230 in H5-V (H1 HA numbering). The substitution of D193 by glutamate has been correlated with a switch from α2,3- to α2,6-linked sialic acid [32], which is consistent with the results for H1 and H5-V HA. Interestingly, H5-Q and H9 exhibit dual binding with E and D, respectively, at position 193.

**Figure 4:**
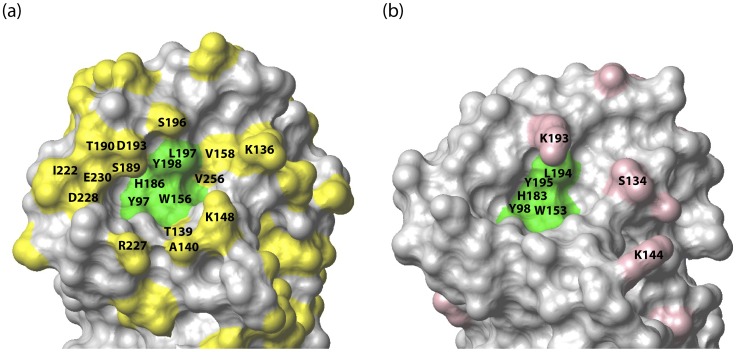
Structural determinants of HA receptor preference. (a) Surface topology of the sialic acid binding site on H1 HA [33] (PDB entry 3AL4). The side chains of non-conserved residues between H1 and H5-V HA are colored yellow. (b) Surface topology of the sialic aicd binding site on H5-V HA [31] (PDB entry 2FK0). The side chains of non-conserved residues between H5-V and H5-Q are colored red. In both (a) and (b) the side chains of residues that form interactions with sialic acid [30] are colored green. The structures shown for H1 and H5-V were determined in the absence of receptor analog (there are no available structures for HA-receptor complexes of these particular HA; however, there are structures for the HA-receptor complexes of other HA strains [7]).

It is next of interest to consider structural differences between H1 and H5-V, for which high-resolution structural data exists [31], [33], and the potential determinants of receptor preference. The overall fold of the two HA is very similar as evidenced by the backbone RMSD of 0.9 Å and the nearly identical placement of the five conserved residues implicated in sialic acid contact ([30], Figure S3). In Figure 4, the amino acid sequence differences between H1 and H5-V HA are colored yellow (the numbering corresponds to that of H1 A/California/04/09 HA1). For reference, the side chains of the conserved receptor binding site contact residues (Y97, W156, H186, L197, and Y198, 14) are colored green. The substituted residues that are in closest proximity to the receptor binding site include K136, T139, A140, K148, V158, S189, T190, D193, S196, I222, R227, D228, E230 and V256. Of these, T139, D193, S196, and E230 of H1 HA have been discussed above. In contrast residues E219 and P224, which were previously implicated in receptor preference [31], are relatively distant from the receptor binding site in the structure of H1 HA. We suggest that the remaining substituted residues (K136, A140, K148, V158, S189, T190, I222, R227, D228 and V256) may represent novel factors in receptor preference. It is next of interest to compare H5-V HA to that of H5-Q. As discussed above, H5-V exhibits preference for α2,3-linked sialic acid while H5-Q binds to both sialic acid with similar affinity. In Figure 3, the amino acid differences between these two isoforms are shaded red. In total there are 14 differences in the sequence. In Figure 4b, we map the sequence differences onto the structure of H5-V with the receptor binding site shown in green for reference. Interestingly, there are only three substitutions whose residues are in close proximity to the receptor binding site: S134D, K144R and K193R. As a consequence, one or a combination of substitutions must be responsible for the change in receptor specificity (there are no substitutions to non-exposed residues). Notably residue 193, which lies just above the receptor binding site, has been implicated in receptor specificity (discussed above, cf. [31]), however, in the present case the lysine to arginine change is relatively conservative. Moreover, to our knowledge positions 134 and 144 have not been previously implicated in receptor preference. Taken together, the amino acid sequence alignment and x-ray structures suggest that receptor preference may be driven by numerous possible changes, which on an idividual basis may be relatively minor. Interestingly, the small differences in HA structure are consistent with our observation of subtle differences in binding mode (Table 1).

### Conclusions

In the present study we have used STD NMR to establish the relative affinity of HA from three different subtypes for receptor analogs. We found three phenotypes: H1 HA with preference for α2,6-linked sialic acid, H5-V with preference for α2,3-linked sialic acid, and H5-Q and H9 with similar affinity for both sialic acid linkages. Importantly, we also found that each subtype binds to both receptors, which may allow influenza strains to gain a foothold in species with a population of different receptors. Therefore, not only receptor preference but also the overall receptor binding profile of a particular HA subtype may give valuable insight into host tropism, as well as other factors such as fucosylation, sulfation, sialylation and host factor interactions [28], [31]. Finally, we note that the STD NMR experiment may be used to quickly determine the binding profiles of newly discovered HA, which in the context of receptor usage, is of utmost interest. Indeed, Haselhorst et al. [19] have recently shown that virus like particles containing H5 HA show preferential binding to 3′SL using the STD NMR competition assay.

## Materials and Methods

The HA samples from subtypes H1 A/California/04/09 (H1N1), H5 A/bar-headed goose/Qinghai/1A/05 (H5N1), H5 A/Vietnam/1205/04 (H5N1), and H9 A/Hong Kong/1073/99 (H9N2) were obtained from BEI Resources (Manassas, VA). In each case the HA are full length, prepared in cell culture, and glycosylated. The purity of each HA was verified by SDS-PAGE. The sialic acid receptor analogs, 2,3-sialyllactose (3′SL) and 2,6-sialyllactose (6′SL), were obtained from V-Labs (Covington, LA). For the STD experiments, the experimental conditions were 3 mM sialic acid and 2 µM (monomer) of the respective HA in PBS buffer (pH 7.4). STD NMR experiments were performed with a train of 50 msec gaussian-shaped saturating pulses at 100 Hz power for 3 sec with “on” resonance saturation at −0.4 ppm and “off” resonance saturation at 30 ppm (the relaxation delay was 2 sec before the saturating pulses). The number of scans was 1728 and the spectral width was 14,367 Hz. Spectra were recorded at 25°C on a Bruker AVANCE 900 MHz spectrometer equipped with a cryogenic probe. Spectra were processed by NMRPipe with a 5 Hz line broadening and analyzed by NMRDraw [14]. %STD was defined as 100×ΔI/I_off_ where ΔI = I_off_-I_on_ and I_off_ and I_on_ are the intensities observed for the various resonances after the “off” and “on” presaturation of HA. Errors in the %STD were estimated as ΔI/I_off_((N_ΔI_/ΔI)∧2+(N_Ioff_/I_off_)∧2)∧0.5 [15], where N_ΔI_ and N_Ioff_ are the noise calculated by NMRDraw in the appropriate spectrum. We note that the STD experiment can be used to determine K_d_ in certain cases [16]; however, our attempts to measure K_d_ by titration failed due to additional binding of sialic acid at concentrations greater than 5 mM, as manifested by an inability to achieve ligand saturation.

## Supporting Information

Figure S1 **STD NMR spectrum of 3′SL and 6′SL in the absence of HA (the control experiment for the STD).** The experimental conditions were 3 mM SA in PBS (pH 7.4) at 25°C. Note that these spectra were obtained on a Bruker 800 MHz spectrometer equipped with a room temperature triple resonance probe.
(PDF)

Figure S2 **STD competition assay for H5-Q binding to 3′SL and 6′SL.** The experimental conditions were 2 uM HA, 3 mM 6′SL, 3 mM 3′SL (upper spectrum) or 6 mM 3′SL (lower spectrum) in PBS, pH 7.4 at 25°C.
(PDF)

Figure S3 **Structural alignment of influenza HA H1 (red) and H5-V (blue) backbones.** The green residues correspond to residues that interact with SA.
(PDF)

Table S1 **% STD intensity for HA-SL interactions^1^.**
(PDF)

Materials and Methods S1 (PDF)
