# Supplementary material for: Characterization of Influenza Hemagglutinin Interactions with Receptor by NMR
Source: PLoS One. 2012 Jul 16;7(7):e33958. doi: 10.1371/journal.pone.0033958 (PMC3397988; doi:10.1371/journal.pone.0033958)

Figure S1: STD NMR spectrum of 3'SL and 6'SL in the absence of HA (the control experiment for the STD). The experimental conditions were 3 mM SA in PBS (pH 7.4) at 25˚C. Note that these spectra were obtained on a Bruker 800 MHz spectrometer equipped with a room temperature triple resonance probe.

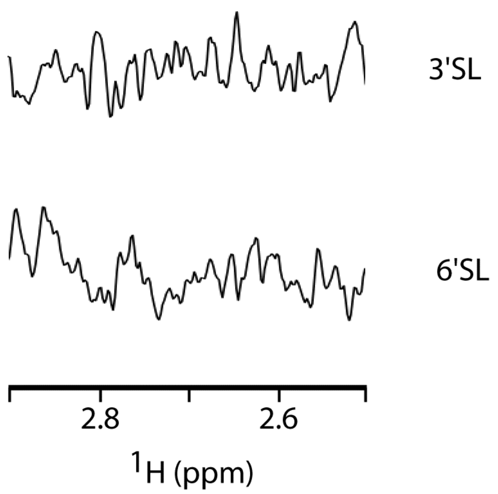

Supplement: Figure S1 — STD NMR spectrum of 3′SL and 6′SL in the absence of HA (the control experiment for the STD). The experimental conditions were 3 mM SA in PBS (pH 7.4) at 25°C. Note that these spectra were obtained on a Bruker 800 MHz spectrometer equipped with a room temperature triple resonance probe. (PDF) [file pone.0033958.s001.pdf]
