# Supplementary material for: Characterization of Influenza Hemagglutinin Interactions with Receptor by NMR
Source: PLoS One. 2012 Jul 16;7(7):e33958. doi: 10.1371/journal.pone.0033958 (PMC3397988; doi:10.1371/journal.pone.0033958)

Figure S2: STD competition assay for H5-Q binding to 3'SL and 6'SL. The experimental conditions were 2 uM HA, 3 mM 6'SL, 3 mM 3'SL (upper spectrum) or 6 mM 3'SL (lower spectrum) in PBS, pH 7.4 at 25˚C.

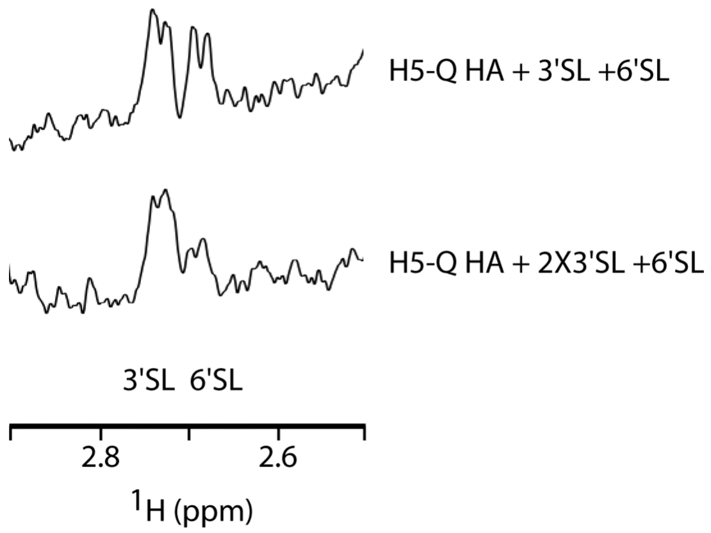

Supplement: Figure S2 — STD competition assay for H5-Q binding to 3′SL and 6′SL. The experimental conditions were 2 uM HA, 3 mM 6′SL, 3 mM 3′SL (upper spectrum) or 6 mM 3′SL (lower spectrum) in PBS, pH 7.4 at 25°C. (PDF) [file pone.0033958.s002.pdf]
