# Supplementary material for: Characterization of Influenza Hemagglutinin Interactions with Receptor by NMR
Source: PLoS One. 2012 Jul 16;7(7):e33958. doi: 10.1371/journal.pone.0033958 (PMC3397988; doi:10.1371/journal.pone.0033958)

Figure S3: Structural alignment of influenza HA H1 (red) and H5-V (blue) backbones. The green residues correspond to residues that interact with SA.

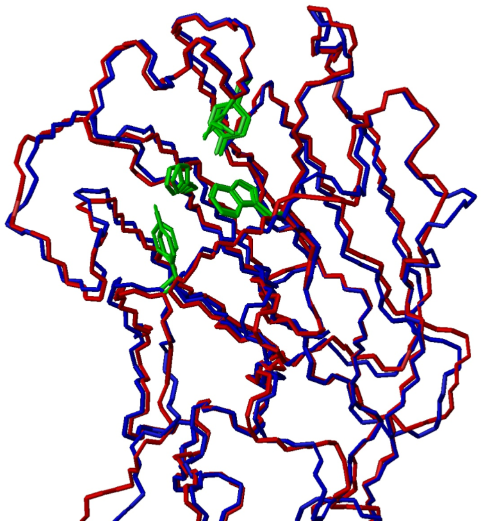

Supplement: Figure S3 — Structural alignment of influenza HA H1 (red) and H5-V (blue) backbones. The green residues correspond to residues that interact with SA. (PDF) [file pone.0033958.s003.pdf]
