# Supplementary material for: Characterization of Influenza Hemagglutinin Interactions with Receptor by NMR
Source: PLoS One. 2012 Jul 16;7(7):e33958. doi: 10.1371/journal.pone.0033958 (PMC3397988; doi:10.1371/journal.pone.0033958)
Supplement: Table S1 — % STD intensity for HA-SL interactions1. (PDF) [file pone.0033958.s004.pdf]

Table S1: % STD intensity for HA-SL interactions[1].

Interaction	H_{3eq} (%)	H_{3ax}(%)	Acetyl (%)
H1:3'SL	0.20+/-0.01	0.22+/-0.01	0.24+/-0.01
H1:6'SL	0.20+/-0.01	0.24+/-0.01	0.33+/-0.01
H5-V:3'SL	3.25+/-0.20	3.21+/-0.19	7.11+/-0.14
H5-V:6'SL	1.27+/-0.08	1.33+/-0.08	4.32+/-0.09
H5-Q:3'SL	0.26+/-0.02	0.94+/-0.06	1.32+/-0.03
H5-Q:6'SL	0.15+/-0.01	0.66+/-0.04	0.30+/-0.01
H9:3'SL	0.52+/-0.03	0.43+/-0.03	1.56+/-0.03
H9:6'SL	0.58+/-0.04	0.60+/-0.04	2.37+/-0.05

[1]The H_{3eq}, H_{3ax} and Acetyl resonances of 3'SL occur at 2.73, 1.77 and 2.00 ppm, respectively. The H_{3eq}, H_{3ax} and Acetyl resonances of 6'SL occur at 2.67, 1.70 and 2.00 ppm, respectively.
