# Supplementary material for: Characterization of Influenza Hemagglutinin Interactions with Receptor by NMR
Source: PLoS One. 2012 Jul 16;7(7):e33958. doi: 10.1371/journal.pone.0033958 (PMC3397988; doi:10.1371/journal.pone.0033958)
Supplement: Materials and Methods S1 — (PDF) [file pone.0033958.s005.pdf]

Supplementary Materials and Methods

The relative ratios for $K_d^{3'SL}/K_d^{6'SL}$ are inversely proportional to the ratio of STD signals in the competition experiment. Briefly, for the equilibria:

$HA + 3'SL \leftrightarrow HA\text{-}3'SL$ and $HA + 6'SL \leftrightarrow HA\text{-}6'SL$

$K_d^{3'SL} = [HA][3'SL]/[HA\text{-}3'SL]$ and $K_d^{6'SL} = [HA][6'SL]/[HA\text{-}6'SL]$

$[HA\text{-}3'SL] = [HA][3'SL]/K_d^{3'SL}$ and $[HA\text{-}6'SL] = [HA][6'SL]/K_d^{6'SL}$

In the concentration range $[SL] < 3X\ K_d$, STD intensity is proportional to HA bound to SL (Meyer B, Peters T (2003) NMR spectroscopy techniques for screening and identifying ligand binding to protein receptors. Angewantde Chemie International Edition 42: 864-890). Accordingly,

$STD^{3'SL}/STD^{6'SL} \sim [HA\text{-}3'SL]/[HA\text{-}6'SL] = ([HA][3'SL]/K_d^{3'SL})/([HA][6'SL]/K_d^{6'SL})$

Since $[3'SL] \sim [6'SL] \sim 3$ mM, $STD^{3'SL}/STD^{6'SL} \sim K_d^{6'SL}/K_d^{3'SL}$
